# Analysis of the effectiveness of combined CT angiography, MMP-9, and PAF testing in the assessment of vascular restenosis in acute coronary syndromes after atorvastatin combined with tirofiban therapy

**DOI:** 10.5937/jomb0-54706

**Published:** 2025-06-13

**Authors:** Jia Sun, Yiling Gao, Jing Ma, Minghui Zhu, Kefei Li, Yiyong Hou, Huan Zhang, Chunqiao Xie

**Affiliations:** 1 Southeast University, Nanjing Tongren Hospital, School of Medicine, Southeast University, Nanjing, China; 2 Nanjing Tianyinshan Hospital, Department of Pharmacy, Nanjing, China; 3 Southeast University, Nanjing Tongren Hospital, School of Medicine, Department of Cardiology, Nanjing, China

**Keywords:** acute coronary syndrome, computed tomography angiography, coronary angiography, matrix metalloproteinase 9, platelet-activating factor, akutni koronarni sindrom, kompjutervizijska tomografska angiografija, koronarna angiografija, matriks metaloproteinaza 9, faktor aktivacije trombocita

## Abstract

**Background:**

Acute Coronary Syndrome (ACS) is a very common cardiovascular disease in clinical practice with a very high risk of death. In this study, we observed the effectiveness of CT angiography (CTA), matrix metalloproteinase-9 (MMP-9) and platelet-activating factor (PAF) in the assessment of ACS.

**Methods:**

A total of 124 patients with ACS admitted to our hospital from June 2022 to March 2024 were enrolled as study subjects. All study subjects were examined using coronary angiography (CAG) and CTA. To compare the detection rate of ACS by CAG and CTA and the difference in effectiveness in assessing coronary plaque stenosis and plaque calcification. In addition, the levels of MMP-9 and PAF were detected in the patients to analyze their relationship with the degree of stenosis and plaque grade. Subsequently, the effect of the three combined tests, CTA, MMP-9 and PAF, was analyzed to assess postoperative vessel restenosis. Finally, we examined the factors associated with poor CTA image quality.

**Results:**

There was no difference in the effectiveness of CTA in detecting ACS, assessing coronary plaque stenosis and plaque calcification compared with CAG (P>0.05). There was a positive correlation between MMP-9, PAF and the degree of coronary stenosis and plaque severity (P<0,05). The diagnostic accuracy of the combination of CTA, MMP-9, and PAF in diagnosing stenosis in postoperative ACS was 97.58% (Kappa=0.946). It was determined that 124 patients and 21 patients had poor CTA images, and logistic regression analysis showed that shorter breath-hold time and arrhythmia were independent risk factors for poor CTA image quality.

**Conclusions:**

CTA, MMP-9 and PAF are excellent for diagnosing stenosis after ACS, and shorter breath-hold time and arrhythmia are all independent risk factors for poor CTA image quality.

## Abstract (sr)

**Uvod:**

Akutni koronarni sindrom (ACS) je veoma česta kardiovaskularna bolest u kliničkoj praksi sa vrlo visokim rizikom od smrtnog ishoda. U ovom istraživanju, posmatrali smo efikasnost CT angiografije (CTA), matriks metaloproteinaze-9 (MMP-9) i faktora aktivacije trombocita (PAF) u proceni ACS.

**Metode:**

U istraživanju je učestvovalo ukupno 124 pacijenata sa ACS koji su primljeni u našu bolnicu od juna 2022. do marta 2024. godine. Svi ispitanici su podvrgnuti koronarnoj angiografiji (CAG) i CT angiografiji (CTA). Cilj je bio da se uporedi stopa detekcije ACS pomoću CAG i CTA, kao i razlika u efikasnosti u proceni stenoze koronarnog plaka i kalcifikacije plaka. Pored toga, mereni su nivoi MMP-9 i PAF kod pacijenata kako bi se analizirala njihova povezanost sa stepenom stenoze i razredom plaka. Nakon toga, analiziran je efekat tri kombinovana testa, CTA, MMP-9 i PAF, na procenu postoperativne restenoze krvnih sudova. Na kraju, ispitani su faktori povezani sa lošim kvalitetom CTA slika.

**Rezultati:**

Nije bilo razlike u efikasnosti CTA u detekciji ACS, proceni stenoze koronarnog plaka i kalcifikacije plaka u poređenju sa CAG (P>0,05). Postojala je pozitivna korelacija između MMP-9, PAF i stepena koronarne stenoze i ozbiljnosti plaka (P<0,05). Dijagnostička tačnost kombinacije CTA, MMP-9 i PAF u dijagnostikovanju stenoze u postoperativnom ACS bila je 97,58% (Kappa=0,946). Utvrđeno je da je 124 pacijenata imalo loše CTA slike, dok je 21 pacijent imao loš kvalitet slika. Logistička regresiona analiza je pokazala da su kraće vreme zadržavanja daha i aritmija nezavisni faktori rizika za loš kvalitet CTA slika.

**Zaključak:**

CTA, MMP-9 i PAF su izuzetni za dijagnostikovanje stenoze nakon AKS, a kraće vreme zadržavanja daha i aritmija su nezavisni faktori rizika za loš kvalitet CTA slika.

## Introduction

Acute coronary syndrome (ACS) is a clinical syndrome caused by acute myocardial ischemia, which is characterized by rapid onset and progression and poses a significant health threat to patients [1]. According to statistics, the number of new cases of ACS worldwide has exceeded 30,000,000 by 2020, about 2–3 times higher than that in 2010 [2]. In particular, the prevalence of ACS is about 8.4% in people over 60 [3]. As a high-risk disease, ACS carries an extremely high risk of death, with a 5-year mortality of up to 40% [4]. Therefore, the diagnosis and treatment of ACS are significant in ensuring a healthy prognosis for patients. Coronary stenting (CS) is the main option for treating ACS in the clinic, and its clinical application value has been verified many times [5]. Whether the procedure can accurately accomplish the patient’s vascular patency and remove all calcified plaques determines the patient’s prognosis [6]. Therefore, imaging is needed in the clinic to assess the vascularization of ACS patients before CS.

Coronary angiography (CAG) is essential for diagnosing ACS with high accuracy. It is currently recognized as the most accurate diagnostic tool and is used as the »gold standard« [7]. However, CAG is challenging to administer, entails a heightened risk of trauma, is not well-tolerated by certain patients, and carries a substantial cost, thereby restricting its utilization [8]. CT angiography (CTA) is a noninvasive and rapid diagnostic technique that enables physicians to assess coronary artery stenosis and plaques utilizing three-dimensional reconstruction technology to visualize the vascular status [9]. In recent years, several studies have proposed that CTA can also realize accurate ACS assessment [10] [11], but there is still a lack of authoritative reports to confirm this.

On the other hand, post-CS vascular restenosis is also one of the important aspects of ACS treatment that should not be ignored [12]. Statistics show that the likelihood of vascular restenosis in ACS patients after CS is 10–20% [13]. And postoperative vascular restenosis is not only a direct indicator for assessing the success of CS surgery, but also a key to determine the prognosis of patients’ recovery [14]. Therefore, how to realize rapid and accurate assessment of vascular restenosis is also an important problem that needs to be solved urgently in the clinic.

On the other hand, matrix metalloproteinase-9 (MMP-9) and platelet-activating factor (PAF) are recognized as important cardiovascular lesion assessment potential indicators [15]. MMP-9, as a member of the matrix metalloproteinase family, is a well-recognized marker of inflammatory injury in cardiovascular and has also been shown to promote apoptosis, fibre rupture, and inhibition of collagen synthesis in vascular smooth muscle, which is key to the progression of ACS [16]. Conversely, PAF is a phosphate mediator that promotes inflammatory effects, activates platelet-aggregation capacity, and accelerates plaque formation and expansion [17]. However, although MMP-9 and PAF are considered to have excellent potential for ACS assessment, it is also difficult to accurately identify ACS because of their hypersensitive response to various types of inflammatory injury [18] [19]. In this regard, we considered a new protocol for ACS assessment, a three-item combined test for CTA, MMP-9 and PAF. We believe that MMP-9 and PAF can improve the early diagnosis rate of ACS. At the same time, the diagnostic specificity of CTA can complement the limitations of MMP-9 and PAF, thus realizing the ideal ACS diagnosis. However, there is still a lack of reports to support this view.

On the other hand, although other studies have analyzed the application of CTA in ACS, we found that these studies mainly focused on the assessment effect of CTA on ACS, and very few studies have explored the relevant factors that affect poor CTA images. We contend that analysing the factors contributing to suboptimal CTA images can provide a more reliable foundation for future clinical practice, particularly in CTA examinations on patients with ACS.

In this study, we analyze the effect of CTA+MMP-9+PAF on the disease assessment of ACS and the risk assessment of vascular restenosis after CS and further explore the related factors affecting image quality to provide more reliable references for the diagnosis and treatment of ACS in the future.

## Materials and methods

### Sample size calculation

Set the confidence interval =95%, statistic (Z)=1.96, error (E)=10%, probability (P)=0.5, and calculate the sample size (N)=P×(1-P)×Z^2^/E^2^=96.

### Material collection

A total of 124 patients with ACS admitted to our hospital from June 2022 to March 2024 were enrolled as study subjects for retrospective analysis. CAG and CTA examined all study subjects. The study has been approved by the Ethics Committee of our Hospital (NO.TRLLKY2021016), and all the subjects above have been informed and signed the informed consent form.

### Inclusion and exclusion criteria

Inclusion criteria: Patients diagnosed with ACS by electrocardiogram and coronary angiography; patients aged >18 years old; patients who were conscious without speech or communication disorders; treatment-naive patients; patients with complete clinical data; patients who agreed to cooperate with the arrangements of the medical staff of the hospital; patients with high compliance. Exclusion criteria: patients with severe liver or renal insufficiency; patients with other cardiovascular and cerebrovascular diseases; patients with hematologic or endocrine disorders; patients with serious infectious diseases; patients with tumours.

### Imaging inspection

CTA (64-row spiral CT SOMATOM Emotion, Siemens, Germany) utilized a working voltage set at 120 kV, current of 120 mA, pitch of 0.20 mm, scanning layer thickness of 0.625 mm, matrix size 512×512, scanning time ranging from 5 to 9 seconds, and rotational speed of 0.352 revolutions per second. An iodine allergy test was conducted before the examination, and the patient’s heart rate was monitored. If the heart rate exceeded 85 beats per minute, medication was administered to lower it, ensuring a stable heart rate below 85 beats per minute for the commencement of the scan. The patient was positioned supine while the scan covered the thoracic inlet to the diaphragm. Contrast agent iodixanol injection (Yangzijiang Pharmaceutical Group Co., Ltd., H20184002) was administered through the elbow vein using a high-pressure syringe (CT motion, Oolich Medical Supplies Co., Ltd.) to scrutinize coronary artery lesions for plaque severity and stenosis, aiming to capture detailed images. Post-examination, image data were uniformly transferred to the workstation for processing employing surface reconstruction technology, multiplanar reorganization technology, volume reproduction technology, and volume reconstruction technology to generate both two-dimensional and three-dimensional reproductions of coronary arteries, eliminating any blood pool interference.

**Figure 1:**
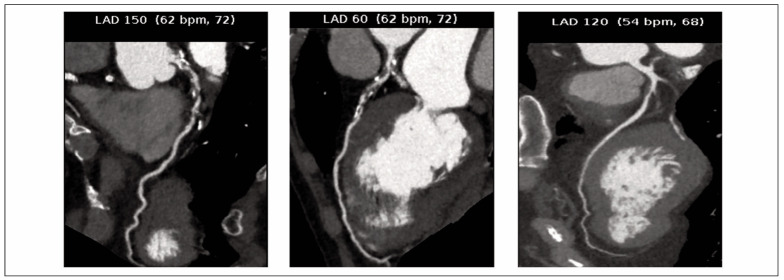
CAG of a typical case. A mixed plate with moderate-to-severe luminal stenosis in the middle wall of the left anterior descending branch. Coronary angiography (CAG).

CAG involved puncturing the patient’s femoral artery for catheter insertion into the left and right orifices of the coronary artery. The left coronary artery was examined initially, followed by the right coronary artery post-contrast medium injection, resulting in four images captured for each artery. The patient was also positioned at two angles to collect image data. Two senior imaging physicians evaluated all image outcomes using a double-blind reading method. Figure 1 demonstrates the imaging findings of a patient with a typical case of ACS.

### Blood sample collection and testing

Three mL of fasting elbow vein blood was collected from patients at admission to the hospital in procoagulant tubes. The serum samples were centrifuged after 30 min of standing at room temperature, and then MMP-9 and PAF were detected using the Enzyme-Linked Immunosorbent Assay (ELISA) method. Kits were purchased from Hangzhou Lianke Bio-technology Co.

### CTA image quality assessment

The same imaging physician reviewed the scans from the two groups of patients, evaluating the quality of the images obtained from the scans per the coronary artery image assessment criteria developed by the American College of Cardiology [20]. Image quality was evaluated based on clarity, lesion visibility, and impact on final outcomes. An image was categorized as good when it displayed clear coronary vessels, stenosis, plaques, and other lesions. Conversely, images with artefacts obscuring arteries in regions of interest (ROI) and affecting tissue clarity were deemed poor.

### Surgical approach

The same surgeon who performed the CS procedure in our hospital treated the patients. Using the coronary artery as the main trunk port and the direction of the catheter as the basis, a guidewire was passed through the lesion and the distal end of the vessel as the endpoint, and the balloon was delivered along the guidewire into the vessel where the lesion occurred. After the balloon reaches the lesion, pressure is applied to complete vasodilation. The balloon was withdrawn after dilatation to achieve the desired effect, and a balloon catheter with a stent was inserted along the guidewire to the lesion location. The stent was opened after filling the balloon to realize balloon decompression. One month after the procedure, CAG and CTA were performed again to confirm vascular patency after the CS procedure.

### Outcome measures

(1) Coronary artery lesion detection: including the left main trunk, left anterior descending branch, left circumflex branch and right coronary artery. (2) Degree of coronary artery stenosis: mild stenosis was shown as <50%, moderate stenosis as 50% to <75%, and severe stenosis as 75%. (3) Plaque calcification: plaque with smooth surface, broad base, centripetal or eccentric, type I; narrow base with bumpy surface and visible niche shadow, type II; plaque with variable length and narrow base, type III. (4) Factors associated with poor CTA images.

### Statistical methods

The statistical software SPSS 23.0 was used to analyze the results obtained. Enumeration data were expressed as [n (%)], with the chi-square test for comparison. Measurement data were expressed as (x̄ ± s), with the independent sample t-test for inter-group comparison. Correlations were analyzed using Spearman’s correlation coefficient. The diagnostic efficacy of CTA, CAG, and combined tests was calculated using the Kappa test, and when the Kappa value was closer to 1, it indicated a better diagnostic effect. Diagnosis of MMP-9 and PAF was analyzed using Receiver operating characteristic (ROC) curves. The Area under curve (AUC) judged the diagnostic effect, and the closer the AUC was to 1, the better the diagnostic effect. Diagnostic compliance was tested using the Kappa test, with a higher Kappa indicating a higher reference value, and correlates were analyzed using Logistic regression. P<0.05 was considered statistically significant.

## Results

### The difference in the detection rate of ACS between CTA and CAG

Table 1 shows that the detection rate of ACS by CTA was 98.39%, and the difference was not statistically significant compared with CAG (*P*>0.05).

**Table 1:** Comparison of detection rate. Note: Coronary angiography (CAG), CT angiography (CTA).

	Left main<br>coronary artery	Left anterior<br>descending artery	Left circumflex<br>artery	Right coronary<br>artery	Detection rate
CAG (n=124)	29 (23.39)	32 (25.81)	30 (24.19)	33 (26.61)	124 (100.00)
CTA (n=124)	28 (22.58)	32 (25.81)	29 (23.39)	33 (26.61)	122 (98.39)
* χ^2^*	0.023	1.000	0.022	1.000	2.016
* P *	0.880	1.000	0.881	1.000	0.156

### Difference in the effectiveness of CTA and CAG in the assessment of ACS

The comparison of coronary plaque stenosis and plaque calcification examination in Table 2 revealed no statistically significant difference between CTA and CAG (*P*>0.05).

**Table 2:** Evaluation of effectiveness.

		CAG (n=124)	CTA (n=124)	χ^2^	P
Coronary artery<br>plaque stenosis<br>status	Mild	65 (52.42)	63 (50.81)	0.065	0.799
	Moderate	46 (37.10)	48 (38.71)	0.069	0.794
	Severe	13 (10.48)	11 (8.87)	0.185	0.668
	Overdiagnosis	-	2 (1.61)	2.016	0.156
Plaque <br>calcification	Type I	64 (51.61)	60 (48.39)	0.258	0.612
	Type II	48 (38.71)	49 (39.52)	0.017	0.897
	Type III	12 (9.68)	13 (10.48)	0.044	0.833
	Overdiagnosis	-	2 (1.61)	2.016	0.156

### Relationship between MMP-9, PAF, and coronary stenosis and plaque severity

**Figure 2:**
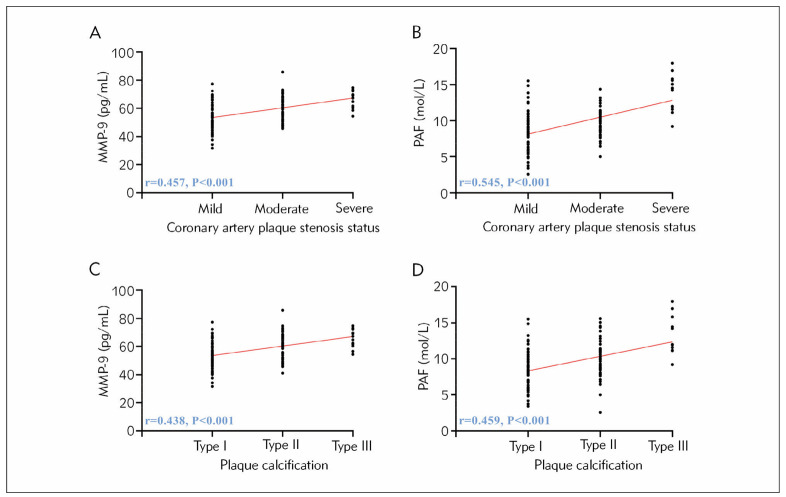
Relationship between MMP-9, PAF, and coronary stenosis and plaque severity. (A) Correlation between MMP-9 and coronary plaque stenosis. (B) Correlation between PAF and coronary plaque stenosis. (C) Correlation between MMP-9 and plaque calcification. (D) Correlation between PAF and coronary plaque stenosis. Matrix metalloproteinase-9 (MMP-9), platelet-activating factor (PAF).

The MMP-9 and PAF of 124 patients were tested to be (57.46±10.17) pg/mL and (9.51±2.88) mol/L, respectively. Spearman’s correlation coefficient analysis found a positive correlation between the levels of MMP-9, PAF, and the degree of coronary artery stenosis and plaque severity (P<0.05, Figure 2). That is, the more stenotic the coronary artery and the more severe the plaque, the higher the levels of MMP-9 and PAF.

### Diagnostic effect of MMP-9 and PAF on postoperative stenosis of CS vessels

**Figure 3:**
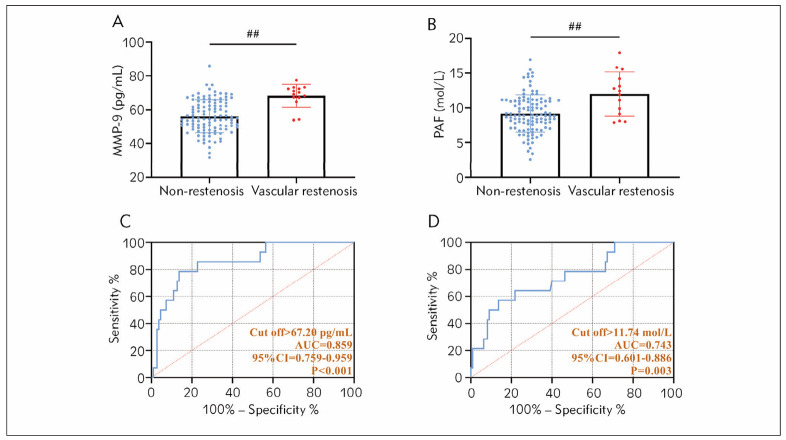
Diagnostic effect of MMP-9 and PAF on postoperative stenosis of CS vessels. (A) Comparison of MMP-9 in patients with restenosis and patients without stenosis after A surgery. (B) Comparison of PAF in patients with restenosis and patients without stenosis after A surgery. (C) Diagnostic value of MMP-9 for postoperative revascularization. (D) Diagnostic value of PAF for postoperative revascularization. ^##^P<0.01. The Area under the curve (AUC) is the confidence interval (CI).

The results of the postoperative examination showed that 14 of 124 patients had restenosis, and the remaining 110 patients’ vessels remained patent. The comparison showed that MMP-9 and PAF were higher in patients with stenosis than those without (P<0.05). By ROC curve analysis, the diagnostic sensitivity and specificity of MMP-9 for postoperative stenosis were 78.57% and 86.36%, respectively (P<0.001). The diagnostic sensitivity and specificity of MMP-9 for postoperative vascular stenosis of PAF were 57.14% and 86.36%, respectively (P<0.001, Figure 3).

### Effectiveness of CTA+MMP-9+PAF in assessing vascular condition after CS surgery

For the three combined CTA, MMP-9, and PAF tests, we used MMP-9 and PAF to reach the cut-off value and use CTA judgment of stenosis as the combined diagnostic criteria. The results showed that CTA+MMP-9+PAF detected 15 cases of postoperative stenosis. Using CAG as a reference, the diagnostic sensitivity, specificity, and accuracy of CTA for diagnosing postoperative vascular restenosis after CS was found to be (Kappa=0.946), respectively, with high reference values (Table 3).

**Table 3:** Effectiveness of CTA in assessing vascular condition after CS surgery. Note: matrix metalloproteinase-9 (MMP-9), platelet-activating factor (PAF)

		CTA+MMP-9+PAF			Kappa	Sensitivity	Specificity	Accuracy
		(+)	(-)	Total				
CAG	(+)	13	2	15	0.946	92.86%	98.18%	97.58%
	(-)	1	108	108				
	Total	14	110					

### Single factor analysis affecting the quality of CTA images

It was determined that of 124 patients, 21 patients had poor CTA images. First of all, we influence the single-factor analysis of poor CTA image. The results show that the difference between the age, gender, and other information of the good and poor image groups is not statistically significant when compared (P>0.05). However, more people with shorter breath-holding time and arrhythmia were in the poor image group than in the good image group (P<0.05, Table 4).

**Table 4:** Single factor analysis affecting the quality of CTA images.

	Good image group<br>(n=103)	Poor image group<br>(n=21)	t or χ^2^	P
Age	60.22±5.98	61.24±4.05	0.743	0.459
Sex			0.067	0.795
Male	62 (60.19)	12 (57.14)		
Female	41 (39.81)	9 (42.86)		
Body mass index (kg/m^2^)	24.51±2.92	25.13±2.57	0.909	0.365
Smoking			0.394	0.530
Yes	32 (31.07)	8 (38.10)		
None	71 (68.93)	13 (61.90)		
Drinking			0.227	0.664
Yes	29 (28.16)	7 (33.33)		
None	74 (71.84)	14 (66.67)		
Breath-holding time			12.180	<0.001
Short	36 (34.95)	16 (76.19)		
Normal	67 (65.05)	5 (23.81)		
Poor contrast filling			0.073	0.788
Yes	8 (7.77)	2 (9.52)		
None	95 (92.23)	19 (90.48)		
Arrhythmia			10.770	0.001
Yes	34 (33.01)	15 (71.43)		
None	69 (66.99)	6 (28.57)		
Severe calcification			1.271	0.260
Yes	45 (43.69)	12 (57.14)		
None	58 (56.31)	9 (42.86)		

### Multifactor analysis affecting CTA image quality

Finally, logistic regression analysis was performed to determine the multifactorial factors affecting the quality of CTA images for the above unifactorial indicators. After assigning values (good image = 1, poor image = 2; normal breath-hold time = 1, shorter breath-hold time = 2; normal rhythm = 1; arrhythmia = 2), the analysis was performed with image quality as the independent variable and the unifactorial indicators as the covariates. The output showed that shorter breath-hold time (OR: 2.542, 95% CI: 1.142–5.443) and arrhythmia (OR: 2.441, 95% CI: 1.114–4.843) were independent risk factors for poor CTA image quality (P<0.05, Table 5).

**Table 5:** Multifactor analysis affecting CTA image quality. Note: Regression coefficient ( ), standard error (S.E.), odds ratio (OR), confidence interval (CI).

	B	S.E	Wald χ^2^	P	OR	95%CI
Breath-holding time	0.942	0.342	5.641	0.018	2.542	1.142–5.443
Arrhythmia	0.547	0.394	5.064	0.025	2.441	1.114–4.843

## Discussion

The incidence of ACS is increasing yearly with the problem of global population ageing [21], which has become one of the main issues endangering the healthy life of patients. In the present study, we observed the application of CTA in assessing ACS.

First, there is no significant difference between the two methods in detecting ACS, stenosis, and calcification. The diagnostic efficacy of both methods is similar, emphasizing the clinical utility of CTA. Although CAG is the most accurate diagnostic modality for ACS, boasting a high accuracy rate, it excels in identifying lesion locations and offering solid grounds for tailoring treatment strategies [22]. CAG is currently the most accurate way to diagnose ACS, with high diagnostic accuracy, which can clarify the location of lesions and provide a reliable basis for developing disease treatment programs [22]. A catheter is inserted through a femoral artery puncture, and a contrast agent is injected into the lesion to obtain a clear image [23]. Doctors can visualize the condition of coronary artery vessels using imaging results to assess the degree of stenosis. Nonetheless, this examination is invasive, posing the risk of causing significant damage to the patient’s blood vessels if not performed correctly. The likelihood of complications is high, and the examination carries a substantial risk, thereby constraining its practical utility [24]. In addition, the high cost of this test is also one of the main reasons affecting its use in clinical practice. Usually, CAG is not performed directly when diagnosing a patient but is generally used when other tests’ results are unclear or cannot be confirmed. Compared with CAG, CTA does not cause trauma to the patient, has a high degree of safety, and requires only the injection of contrast medium through the elbow vein to obtain a clear and rich image [25]. The remarkable spatial resolution of CTA enables physicians to delineate the precise path of the coronary artery, evaluate stenosis severity, and analyze plaque characteristics through image data interpretation. This capability aids in assessing the condition’s severity and formulating or modifying treatment strategies [26]. After the end of scanning, image reconstruction can be completed using various image processing techniques to improve the image quality and help doctors further evaluate the lesions. In the study of Lu ZF et al. [27], CTA can accurately assess the carotid artery stenosis and plaque characteristics. Yang S et al. [28] applied CTA to vertebrobasilar artery detection, which can also accurately assess the stability and vulnerability of intra-arterial plaque. The findings above indicate that CTA is a viable method for investigating the composition of intra-aortic plaque and the extent of arterial stenosis.

On the other hand, we also found a significant correlation between MMP-9 and PAF and ACS patients with coronary stenosis and plaque severity, which again validates the important link between the two chicken ACS. Studies have shown that MMP-9 degrades gelatin, collagen, fibronectin, elastin, etc., which can specifically degrade the extracellular matrix and increase plaque instability [29]. PAF is a plateletassociated factor, and its elevated expression can promote the activation of blood platelets, leading to an increased risk of thrombosis [30]. Therefore, the elevation of both levels often indicates the progression and aggravation of ACS. Thus, we performed a combined test by MMP-9, PAF and CTA and found that the protocol had an excellent diagnostic accuracy of 97.58% for diagnosing postoperative vascular restenosis after ACS. This suggests that in the future, CTA+MMP-9+PAF can be used as an important ACS condition assessment program to improve the clinical treatment of ACS and protect the prognosis of patients. It is well known that vascular patency is the key to ensuring the effect of CS surgery, so it is very important to assess it accurately, which is conducive to timely detection of stenosis and timely treatment to avoid the development of complete occlusion and secondary surgery [31]. CTA has 2 sets of bulb tubes and detection systems with a high spatial and temporal resolution. Its image quality is high, which can clearly display the bridge vessels’ direction and stenosis and avoid the interference of metal artefacts [32]. At the same time, combined with the high sensitivity of MMP-9 and PAF to stenosis and plaque severity, this maximizes the accuracy of the safety assessment after ACS. Meanwhile, as a safe and noninvasive protocol, it can be widely used in hospitals of all levels, further improving the safety and security of ACS treatment.

In this study, we found that 21 of 124 patients had poor CTA images, and logistic regression analysis showed that shorter breath-hold time and arrhythmia were independent risk factors for poor CTA image quality. In this regard, we believe that in the future, when the clinic performs CTA examination on patients, it is necessary to fully communicate with the patients and instruct them on the correct method of breath-holding to avoid the influence of respiratory movement on the images. It is essential to consider that an elevated heart rate or episodes of arrhythmia during CTA can compromise imaging quality and hinder accurate diagnosis, potentially leading to missed diagnoses, as observed in two cases [33] [34]. There fore, managing the patient’s heart rate during the CTA procedure is crucial, aiming to maintain a heart rate of less than 80 beats per minute prior to examination. Additionally, patient cooperation in controlling respiration is necessary to optimize the examination quality. A study by Benson JC et al. [35] proposed that increased calcification of the coronary artery’s inner wall during CTA examinations could negatively impact vessel permeability and contour display, thereby diminishing CTA image quality.

Contrary to these findings, the current study did not observe any impact of plaque calcification on CTA images. This inconsistency may be attributed to the small sample size of patients with severe plaque calcification or the overall limited number of participants in the present study. Consequently, further cases must be included to more rigorously validate the impact of plaque calcification on CTA image quality.

Additionally, the analysis of influencing factors in this study was limited by including a smaller number of indicators. The scope of future studies must be expanded to comprehensively examine the human manipulation and pathological and physiological factors affecting CTA image quality. Of course, the doctor’s professional opinion is an important part of the process for imaging tests. Therefore, we also need to strengthen the professionalism of imaging doctors to reduce the possibility of human misdiagnosis and omission.

## Conclusion

CTA+MMP-9+PAF has an excellent assessment of ACS and can accurately reflect the restenosis of vessels after CS, providing reliable guidance for clinicians. Shorter breath-hold time and arrhythmia are all independent risk factors for poor CTA image quality, and clinical attention needs to be paid to these issues when performing CTA examinations in the future to improve the accuracy of CTA results.

## Dodatak

### Declaration of ethical approval

The study involving human subjects complied with the Declaration of Helsinki and was approved by the ethical committee of the Nanjing Tongren Hospital, School of Medicine, Southeast University (No. TRLLKY2021016), and all participants provided written informed consent.

### Funding

Not applicable.

### Availability of data and materials

The data used to support the findings of this study are available from the corresponding author upon request.

### Acknowledgements

Not applicable.

### Author contributions

Huan Zhang and Chunqiao Xie conceived and designed the project, and Jia Sun and Yiling Gao wrote and modified the manuscript. Jing Ma and Minghui Zhu generated the data. Kefei Li and Yiyong Hou analyzed the data. Jia Sun and Yiling Gao contributed equally to this work as co-first authors. All authors gave final approval of the version to be published and agreed to be accountable for all aspects of the work.

### Conflict of interest statement

All the authors declare that they have no conflict of interest in this work.
